# Composition and fatty acid profile of milk from cows fed diets supplemented with raw and n-3 PUFA-enriched fish oil

**DOI:** 10.1038/s41598-024-61864-z

**Published:** 2024-05-14

**Authors:** Robert Bodkowski, Heliodor Wierzbicki, Anna Mucha, Paulina Cholewińska, Konrad Wojnarowski, Bożena Patkowska-Sokoła

**Affiliations:** 1 Institute of Animal Husbandry and Breeding, Wrocław University of Environmental and Life Sciences, Chełmońskiego 38C, 51-630 Wroclaw, Poland; 2 Department of Genetics, Wrocław University of Environmental and Life Sciences, Kożuchowska 7, 51-631 Wroclaw, Poland; 3 Chair for Fish Diseases and Fisheries Biology, Ludwig-Maximilians-University of Munich, 80539 Munich, Germany

**Keywords:** Environmental sciences, Nutrition

## Abstract

Dietary supplementation of ruminants with fish oil is a strategy for favorably modifying the fatty acid composition of milk fat. This study investigated the effect of supplementing cows’ diet with fish oil after low-temperature crystallisation (LTC-FO) compared to its raw form (FO) on milk yield, milk components (fat, protein, and lactose), and milk fatty acid profile. Twenty-four mid-lactating multiparous Polish Holstein–Friesian cows fed a total-mix ration were randomly assigned to two homogeneous groups (n = 12 cows each) and supplemented with LTC-FO or FO at 1% of dry matter. Milk samples were collected on days 14 and 30 of the 30-day experiment. No significant differences between the groups in terms of milk yield, milk protein, and lactose content were found, however, the fat yield and content decreased in the LTC-FO group. Milk fat from cows in the LTC-FO group contained significantly higher levels of C18:1 *trans*-11, C18:2 *cis*-9, *trans*-11, C18:3*n *− 3, C20:5, and C22:6, and lower levels of saturated fatty acids compared to the FO group (*p* < 0.05). Therefore, LTC-FO may be a more efficient feed additive than FO and may serve as a practical way to modify the fatty acid composition of milk fat.

## Introduction

The demand for foods that may have beneficial effects on consumer health has increased, resulting in a growing interest in strategies to improve the nutritional quality of ruminant-derived products. Consumers are becoming more aware of the relationship between diet and well-being, leading to a growing market for foods with proven health benefits. The content of health-promoting fatty acids (FAs) in milk can be increased effectively through animal feeding strategies^1^.

Omega-3 (*n* − 3, ω − 3) fatty acids, specifically eicosapentaenoic acid (EPA, C20:5) and docosahexaenoic acid (DHA, C22:6), have been shown to be beneficial to human health^2^. The average intake of ω − 3 long-chain polyunsaturated fatty acids (LC-PUFAs) in many European countries is below the recommended level due to the low consumption of fish^3^. Conjugated linoleic acid (CLA, C18:2 isomers) and trans vaccenic acid (TVA, C18:1 *trans*-11) also exhibit biological activity and health-promoting properties^4,5^. The main sources of CLA in the human diet are products from ruminants (milk, meat)^6^.

Cow's milk is a widely used dairy product globally. According to Huth and Park^7^, milk and dairy products contribute around 15% of the total fat and 25% of the saturated fatty acids (SFAs) in Western diets. Cow's milk fat constitutes approximately 2.8–3.7% of PUFAs. However, EPA and DHA are present in small amounts (0.03–0.067 and 0.002–0.03 g/100 g of total FAs, respectively), and CLA represents approximately 0.54–0.74 g/100 g of total FAs^8,9^.

The fat composition of bovine milk depends on the breed, diet and stage of lactation^1,9,10^. Feeding strategies to modulate lipid metabolism and modify the FA composition of cow's milk include supplementing rations with vegetable oils, oilseeds, marine lipids (fish oil, marine algae), using different feed sources (pasture, grass hay and grass silage) and varying the ratio of feed to concentrate^1,11,12^. Fish oil can be added to modify the composition of FAs, for example to increase the content of health-promoting fatty acids in milk, such as EPA, DHA, CLA and TVA^8,13^. Research on the effects of fish oil use in ruminant nutrition on the chemical composition of milk and FA profile has mainly focused on its different doses, administration methods, sources of origin, addition alone or in a combination with vegetable oils^14–16^. In dairy cows, fish oils are usually used in protected form (e.g. saponified in the form of calcium or potassium salts, encapsulated in a matrix of rumen inert proteins, encapsulated in gelatin)^15,17,18^ due to the toxic effects of higher doses on the rumen microbiome and reduced dry matter intake, as well as lower fat content in milk^19,20^. Unprotected form is used much less frequently^13,16^. Fish oil can be used in rations as a component of the basal diet or “top dressing”^13,16^. Experiments on cows indicate that it can also be applied directly to the rumen^8,21^, or to bypass ruminal biohydrogenation as abomasal infusion or duodenal supply^21,22^.

The primary determinant of the biological value of fish oil is the concentration of omega-3 LC-PUFAs. Several techniques can increase the content of *n* − 3 FAs, but only a few are cost-effective and suitable for large-scale production. In the present study a non-complicated and not requiring special equipment low-temperature crystallisation (LTC) process was used. As indicated by an earlier study by the authors of this paper, this method results in a significant increase in the concentration of long-chain PUFA in fish oil^23^. Compared to raw fish oil, the application of an analogous amount of oil after the LTC process results in an increase in the LC-PUFA content of the diet and, consequently, a higher intake by cows.

The aim of this study was to investigate the effect of supplementing dairy cow diets with fish oil enriched with unsaturated fatty acids (UFAs), including *n* − 3 LC-PUFAs resulting from a low-temperature crystallisation process (LTC-FO) compared to its raw form (FO), on milk production, milk ingredient yield and content, FAs composition, and the proportion of FA groups with varying degrees of saturation and carbon chain length. We hypothesised that dietary supplementation of cows with LTC-FO would increase the content of biologically active fatty acids such as EPA, DHA, CLA and TVA in milk fat more than the addition of FO.

## Material and methods

The animals were treated in accordance with the guidelines of the Polish regulations on the conditions for keeping farm animals. The protocol for the animals used in the experiment and the experimental procedures were in accordance with the European Council Directive (86/609/EEC) and the Polish Law (Dz. U. 2015 poz. 266). The 2nd Local Ethical Committee for Experiments on Animals in Wroclaw (Poland) approved the experimental protocols presented in this study (Decision no. 61/2011). The study was reported in accordance with the ARRIVE guidelines.

### Process of low-temperature crystallisation of fish oil

**Figure 1:**
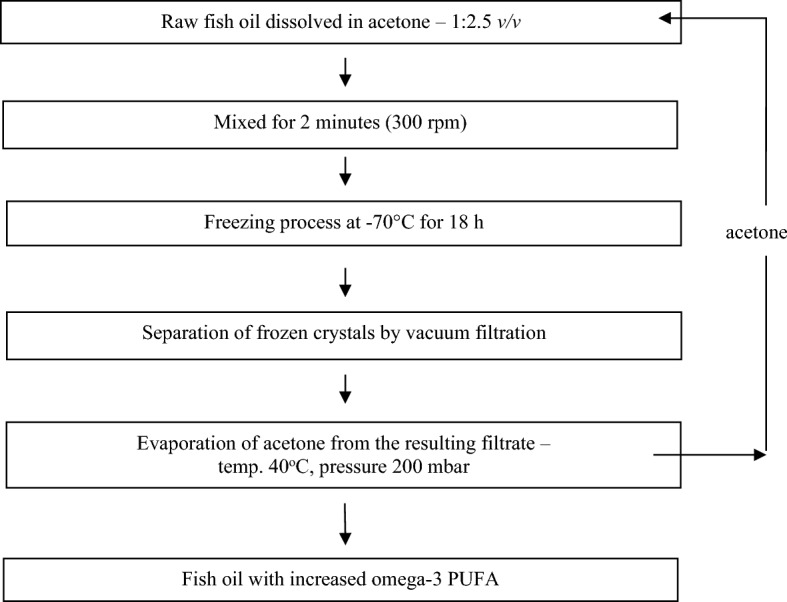
Scheme of the process of low-temperature crystallisation of fish oil.

Raw fish oil was obtained during the production of fish meal (mainly herring and sprat from the Baltic Sea). The concentration of *n* − 3 PUFAs in fish oil was increased by low-temperature crystallisation (LTC)^24^, according to a modified version of the method developed by Bodkowski et al.^23^ (Fig. 1). To protect the unsaturated fatty acids (UFAs) in the raw fish oil and after the LTC process from oxidation, α-tocopherol was added at 200 mg/l and the preparations were stored under refrigerated conditions in closed dark bottles^25^. Chromatographic analysis of fatty acid methyl esters (FAME) of raw fish oil and after the LTC process was performed on an Agilent Technologies 6890N gas chromatograph (Agilent Technologies, Santa Clara, CA, USA) with flame ionisation detector (FID) and SP-2560 capillary GC column (100 m length × 0.25 mm inner diameter (i.d.), d_f_ = 0.20 μm; Supelco, Bellefonte, PA, USA). Details of the LTC process, conditions and temperature for chromatographic analysis and FAME standards have been described elsewhere^26^. The LTC process and chromatographic analysis were performed at the Department of Food and Environmental Chemistry, National Marine Fisheries Research Institute in Gdynia, Poland. Table 1 presents the fatty acid profile of raw fish oil and fish oil after a low-temperature crystallisation process based on six different batches for each additive (mean ± sd).

**Table 1:** Fatty acids ($\overline{x }$ ± sd) in raw (FO) and after the process of low-temperature crystallisation (LTC-FO) sprat-herring oil (g/100 g of total FAs).

Fatty acids	Fish oil	
	FO	LTC-FO
C12:0	0.11^A^ ± 0.04	0.04^B^ ± 0.20
C14:0	6.34^A^ ± 1.16	2.72^B^ ± 0.42
C14:1	0.47^A^ ± 0.14	0.13^B^ ± 0.03
C15:0	0.42^A^ ± 0.16	0.17^B^ ± 0.05
C16:0	15.43^A^ ± 2.12	2.67^B^ ± 0.48
C16:1	7.53^A^ ± 1.33	3.36^B^ ± 0.56
C17:0	0.27^A^ ± 0.07	0.11^B^ ± 0.03
C17:1	2.48^a^ ± 0.44	1.62^b^ ± 0.32
C18:0	2.52^A^ ± 0.39	0.68^B^ ± 0.12
C18:1	24.33 ± 3.31	21.24 ± 2.89
C18:2	4.26 ± 1.26	4.36 ± 1.35
C18:3*n*-3	3.83 ± 0.76	3.93 ± 0.48
C18:3	0.14^a^ ± 0.05	0.10^b^ ± 0.03
C18:4*n*-3	2.23^a^ ± 0.69	2.78^b^ ± 0.54
C20:0	0.12 ± 0.05	0.08 ± 0.03
C22:0	0.13^a^ ± 0.04	0.10^b^ ± 0.03
C20:1	2.45 ± 0.69	3.05 ± 0.54
C20:2	1.14^A^ ± 0.28	2.38^B^ ± 0.42
C20:3	0.15^A^ ± 0.03	0.25^B^ ± 0.05
C20:3*n*-3	0.43^A^ ± 0.06	0.86^B^ ± 0.08
C20:4	0.68^A^ ± 0.11	0.97^B^ ± 0.15
C20:5*n*-3	6.46^A^ ± 1.45	12.26^B^ ± 1.78
C22:1	0.33^a^ ± 0.07	0.47^b^ ± 0.11
C22:5*n*-3	1.82^A^ ± 0.35	3.26^B^ ± 0.40
C22:6*n*-3	14.48^A^ ± 2.41	30.57^B^ ± 3.62
C24:1	0.39^a^ ± 0.11	0.51^b^ ± 0.14
Item^1^		
SFA	25.34^A^ ± 2.98	6.57^B^ ± 1.98
UFA	73.78^A^ ± 2.56	92.27^B^ ± 3.02
MUFA	38.16^A^ ± 2.77	30.55^B^ ± 2.56
PUFA	35.62^A^ ± 2.32	61.72^B^ ± 3.13
LC-PUFA	28.58^A^ ± 1.87	54.76^B^ ± 2.62
Σ *n*-3	29.25^A^ ± 1.76	53.66^B^ ± 2.44
EPA + DHA	20.94^A^ ± 1.93	42.83^B^ ± 2.55

^A,B^Different superscripts indicate significant differences between means in rows at *p* < 0.01.

^a,b^Different superscripts indicate significant differences between means in rows at *p* < 0.05.

^1^SFA—saturated fatty acids; UFA—unsaturated fatty acids; MUFA—monounsaturated fatty acids; PUFA—polyunsaturated fatty acids; LC-PUFA—long-chain polyunsaturated fatty acids (PUFA with twenty or more atoms of carbon); Σ *n*-3—sum of C18:3 *cis*-9,12,15, C18:4 *cis*-6,9,12,15, C20:5 *cis*-5,8,11,14,17, C22:5 *cis*-7,10,13,16,19, and C22:6 *cis*-4,7,10,13,16,19; EPA + DHA—sum of eicosapentaenoic acid (C20:5) and docosahexaenoic acid (C22:6).

### Experimental designs

Animals were selected on the basis of parity, stage of lactation, milk yield and live body weight. Twenty-four multiparous (3–4 lactation) Polish Holstein Friesian cows of the red-white variety (mean ± sd; body weight 612.8 ± 26.3 kg; days in milk 95 ± 21 d; daily milk yield 33.1 ± 3.93 kg) were housed in a tie-stall barn and randomly assigned to the two dietary groups (n = 12 animals each): FO—group received raw fish oil at 1% of dry matter (DM); LTC-FO—group received fish oil after the process of low-temperature crystallisation at 1% of DM. Diets were administered twice daily as a total mixed ration (TMR). Feed additives, i.e. FO and LTC-FO, were nozzle-sprayed onto the natural humus-mineral carrier Humokarbowit (20% of additive/kg carrier; PHW Tronina, Raków, Poland) and applied as a "top dressing". Prior to the start of the experiment, all cows were fed the same TMR with Humokarbowit (without added fish oil) for a two-week adaptation period. During the 30-day experiment the supplements (Humokarbowit with FO or LTC-FO) were administered in the morning at 05:00. Measurements of milk components and FA composition in both groups of cows were taken on days 14 and 30 of the experiment. The cows were milked twice a day (06:00; 18:00).

### Components and nutritional value of cow diets

In both groups studied, the diet was isoproteic and isoenergetic and was formulated to meet the nutritional requirements of dairy cows in mid-lactation according to the INRA feeding system^27^. Samples of the components and TMR were analysed three times during the experiment (at the beginning and after 10 and 20 days) for DM (method 934.01), crude protein (method 984.13; Kjeltec 2300 apparatus, Foss Analytical, Hillerød, Denmark) and crude fat (method 920.39) according to AOAC^28^, and for neutral detergent fiber (NDF) and acid detergent fiber (ADF) according to the method of Van Soest et al.^29^ with the addition of sodium sulphite and amylase, respectively (Ankom200 Fibre Analyser, ANKOM Technology, Macedon, NY, USA). The composition and nutritional value of the TMR and the diet supplemented with LTC-FO or FO are shown in Table 2. In both groups cows received a daily ration containing 24.5 kg DM and the refusal rate did not exceed 7%. All cows had free access to fresh water.

**Table 2:** Composition and nutritional value of TMR and diet supplemented with LTC-FO or FO.

Item	TMR	Diet supplemented with LTC-FO or FO^1^
Ingredient (% of DM)		
Corn silage	39.4	39.4
Grass silage	25.6	25.6
Fresh spent grain	7.2	7.2
Wet beet pulp	6.6	6.6
Rapeseed meal	5.1	5.1
Soybean meal	3.5	3.5
Second-cut hay	4.6	4.6
Complete mixture	7.2	7.2
Humokarbowit^2^*	–	1.7
Raw or after low-temperature crystallization fish oil*	–	1
Calcium bicarbonate	0.25	0.25
Vitamins and minerals premix^3^	0.45	0.45
Nutritive value (% of DM unless otherwise stated)		
Crude protein	15.37	15.12
Ether extract	3.07	4.09
NDF^4^	36.3	35.5
ADF^5^	25.6	25.2
Ca	0.69	0.72
P	0.35	0.37
NE_L,_^6^ Mcal/kg of DM	1.51	1.57

*Ingredient supplemented as “top dressing”.

^1^FO—raw fish oil; LTC-FO—fish oil after the process of low-temperature crystallisation.

^2^Humic-mineral preparation (PHW Tronina, Raków, Poland).

^3^Fatromix BoW3:1—contains: Ca—150 g, P—50 g, Na—130 g, Mg—50 g, Zn—8000 mg, Cu—1500 mg, Mn—6000 mg, I—120 mg, Se—30 mg, Co—20 mg, vitamin A—1,200,000 IU, vitamin D—16,000 IU, vitamin E—5000 mg, vitamin B,—210 mg, and vitamin H,—80 mg (Fatro Polska Ltd., Kobierzyce, Poland).

^4^NDF—neutral detergent fiber.

^5^ADF—acid detergent fiber.

^6^NE_L_—net energy for lactation.

### Chemical and chromatographic analysis

Individual milk samples (100 mL each) from morning milking were collected after 14 and 30 day of the experiment (immediately cooled to 4 °C and analysed within 4 h). Basic milk parameters, i.e. fat, protein and lactose, were analysed using an Infrared 150 apparatus (Bentley Instruments Inc., Chaska, MN, USA) in the Laboratory of Milk Assessment and Analysis at the Institute of Animal Breeding, Wroclaw University of Environmental and Life Sciences, Poland.

Milk fat for chromatographic analysis was obtained as a result of milk centrifugation and extraction according to the modified procedure of Folch^26^. Identification of FAME was performed in the Laboratory of Chromatography and Meat Analysis, Institute of Animal Breeding, Wroclaw University of Environmental and Life Sciences, Poland (Agilent Technologies 7890A gas chromatograph with FID detector, HP-88 capillary GC column (100 m length × 0.25 mm i.d. × 0.20 μm d_f_; Agilent Technologies, Santa Clara, CA, USA). The oven temperature was initially held at 100 °C for 5 min, increased by 4 °C/min to 140 °C, further increased by 2 °C/min to 240 °C, and the final isotherm held for 5 min. Injection was at 1 μL in split mode (80:1 split ratio), injector temperature 250 °C, detector temperature 270 °C, with helium as the carrier gas. FAME standards (GLC #47885, #47791, #46903, #46904, #46905, #43959, #O5632, #D5679 Sigma-Aldrich Chemie GmbH, Schnelldorf, Germany, #20-1823-7, #20-1826-7, Larodan Fine Chemicals AB Products, Malmö, Sweden) were used for the identification of FAs using Agilent ChemStation software (Agilent Technologies, Santa Clara, CA, USA). All fatty acid composition results are expressed as g/100 g FAs. The results obtained were processed and presented as individual FAs and as the content of FA groups with different degrees of saturation and carbon chain length (SFA—saturated FAs, UFA—unsaturated FAs, MUFA—monounsaturated FAs, PUFA—polyunsaturated FAs, SCFA—short-chain FAs with C4–10, MCFA—medium-chain FAs with C12–16, LCFA—long-chain FAs ≥ C17, LC-PUFA—long-chain PUFA ≥ C20) and as the sum of CLA isomers, trans C18:1 isomers, *n*-3 FAs and EPA + DHA.

### Statistical analysis

The normal distribution of the variables analysed was verified using the Shapiro–Wilk test. The homogeneity of variance in the analysed groups, determined by the type of diet (FO vs. LTC-FO) and the day of measurement (14 d vs. 30 d), was verified using the F-test and Bartlett's test. The statistical significance of the effect of diet and day of measurement was tested using a two-way mixed ANOVA in the *rstatix* package^30^, if the assumptions of normality of distribution were met (a linear model with the effect of diet, day of measurement, and the interaction of diet and day of measurement was used). If the assumptions were not met, non-parametric analysis of variance-type testing with the F1-LD-F1 model was applied using the *nparLD* package^31^. Post hoc statistical analysis was performed using pairwise comparisons between diets (FO vs. LTC-FO) within each measurement day (14 d or 30 d) and pairwise comparisons between measurement days (14 d vs. 30 d) within each diet (FO or LTC-FO). A parametric t-test and a non-parametric Wilcoxon test were used. The effect of diet was tested using a two-sample unpaired test, while the effect of day of measurement was tested using a two-sample paired test. All statistical analyses were performed using the R Project for Statistical Computing^32^.

## Results

### Process of low-temperature crystallisation of fish oil

Table 1 presents the results of a comparison between the content of the fatty acids in FO and LTC-FO. The LTC process of fish oil resulted in a significant increase in several fatty acids, including eicosadienoic acid (EDA, 20:2*n* − 6), dihomo-gamma-linolenic acid (DHGLA, 20:3*n* − 6), eicosatrienoic acid (ETE, C20:3*n* − 3), arachidonic acid (AA, C20:4*n* − 3), eicosapentaenoic acid (EPA, C20:5*n* − 3), docosapentaenoic acid (DPA, C22:5*n* − 3), and docosahexaenoic acid (DHA, C22:6*n* − 3) (*p* < 0.01). In addition, the content of UFA, particularly PUFA, LC-PUFA, and *n* − 3 fatty acids increased, while the content of SFA decreased significantly (*p* < 0.01).

### Dry matter intake, production and chemical composition of milk

Table 3 presents the results that demonstrate the significance of the differences between the mean values of dry matter intake (DMI) and milk traits calculated for the two studied groups of cows (FO group vs. LTC-FO group) at the two sampling times (14 day vs. 30 day). It also shows the significance of the effect of the studied factors, namely diet, time, and diet x time interaction, on DMI and milk traits. On day 14 of the experiment, the LTC-FO-group had a lower DMI compared to the FO-group (*p* < 0.05). For the group receiving LTC-FO, a significant difference was also found between day 14 and 30 of the experiment (*p* < 0.05). The DMI was significantly influenced by diet (D) and sampling time (T) (*p* value 0.036 and 0.043, respectively). The developed feed additive, LCT-FO, did not affect milk yield, lactose yield and content, and protein content in milk compared to FO. However, the addition of LCT-FO to diet resulted in a significant decrease (*p* < 0.05) in fat content (3.64% and 3.68% vs. 3.22% and 3.31% at two consecutive sampling times for FO and LTC-FO, respectively) and fat yield (1.18 kg/d and 1.21 kg/d vs. 1.03 kg/d and 1.07 kg/d at two consecutive sampling times for FO and LTC-FO, respectively). The fat content and yield, as well as protein yield, were significantly influenced by diet type (D) and sampling time (T) (*p* value ranging from < 0.001 to 0.034).

**Table 3:** Dry matter intake, and milk traits ($\overline{x }$) of cows fed two diets at two sampling times (n = 12 animals/group).

Item	FO^1^		FO-LTC^1^		SEM^2^	*p*-value^3^		
	14 d	30 d	14 d	30 d		D	T	D × T
DMI, kg/d	23.2^A^	23.6	22.4^Ba^	23.2^b^	0.10	0.036	0.043	0.593
Milk production, kg/d	32.4	32.8	32.1	32.4	0.15	0.326	0.123	0.716
Milk component								
Fat, %	3.64^A^	3.68^A^	3.22^Ba^	3.31^Bb^	0.04	< 0.001	0.017	0.414
Fat, kg/d	1.18^A^	1.21^A^	1.03^B^	1.07^B^	0.02	< 0.001	0.021	0.921
Protein, %	3.22	3.25	3.17	3.23	0.01	0.127	0.149	0.575
Protein, kg/d	1.04	1.07	1.02	1.04	0.01	0.034	0.025	0.872
Lactose, %	4.74	4.76	4.75	4.71	0.01	0.091	0.396	0.068
Lactose, kg/d	1.53	1.56	1.53	1.53	0.01	0.179	0.239	0.232

^A,B^Different superscripts indicate significant differences between diets within sampling time at *p* < 0.05.

^a,b^Different superscripts indicate significant differences between sampling times within diet at *p* < 0.05.

^1^Diet: FO—supplemented with raw fish oil; LTC-FO—supplemented with fish oil after the process of low-temperature crystallisation.

^2^SEM—standard error of the mean.

^3^*p* value: D—effect of diet (FO vs. LTC-FO); T—effect of sampling time (14 d vs. 30 d); D × T—interaction between diet and time.

### Milk fat fatty acid profile

Table 4 shows that milk fat from cows in the LTC-FO fed group had a significantly higher (*p* < 0.05) mean content of fatty acids C16:1*c9*, C18:1 isomers with configurations *t10*, *c11* and *t11*, C18:2*c9*, conjugated dienes of linoleic acid (CLA) with configurations *c*9*t*11, *t*10*c*12 and *t*11*c*13, isomers C18:3 (*n*-3, *n*-6), C20:1, C20:2, C20:3, C20:4, C20:5, C22:1, C22:5, and C22:6 compared to the FO fed group. In contrast, in the group fed LTC-FO, significantly lower (*p* < 0.05) mean levels of C4:0, C6:0, C8:0, C10:0, C12:0, C16:0, C18:0, C18:1*c9*, C18*t9*, and C22:2, were detected compared to FO fed cows. The statistical analysis shows that the type of diet (D) and sampling time (T) had a significant effect on the content of the vast majority of fatty acids analysed in milk fat (most *p* values < 0.001), while the D × T interaction had a significant effect on the content of a slightly smaller number of fatty acids (half of the total analysed fatty acids, *p* value ranging from < 0.001 to 0.049).

**Table 4:** Fatty acids ($\overline{x }$) in milk of cows fed two diets at two sampling times (n = 12 animals/group).

Fatty acids (g/100 g of total FAs)	FO^1^		FO-LTC^1^		SEM^2^	*p* value^3^		
	14 d	30 d	14 d	30 d		D	T	D × T
C4:0	2.83^Aa^	2.72^Ab^	2.47^B^	2.51^B^	0.03	< 0.001	0.165	0.024
C6:0	1.66^A^	1.63^A^	1.38^B^	1.40^B^	0.02	< 0.001	0.900	0.167
C8:0	1.35^A^	1.32^A^	1.20^B^	1.03^B^	0.03	< 0.001	0.035	0.152
C10:0	2.06^A^	2.19^A^	1.79^B^	1.74^B^	0.04	< 0.001	0.569	0.088
C12:0	3.08^Aa^	2.88^b^	2.67^Ba^	2.87^b^	0.03	< 0.001	0.088	< 0.001
C14:0	9.78	9.85^A^	9.75^a^	9.37^Bb^	0.06	0.050	0.093	0.021
*c*9 C14:1	0.89^A^	0.87	0.84^Ba^	0.90^b^	0.01	0.449	0.013	< 0.001
C16:0	28.5	28.1^A^	28.6^a^	27.2^Bb^	0.11	0.032	< 0.001	0.005
*c*9 C16:1	1.62^Aa^	1.74^Ab^	1.94^B^	2.01^B^	0.03	< 0.001	0.001	0.260
C18:0	13.8^Aa^	13.0^Ab^	12.8^B^	12.6^B^	0.09	< 0.001	< 0.001	0.002
*c*9 C18:1	22.9^A^	23.2^A^	20.5^Ba^	21.2^Bb^	0.18	< 0.001	0.004	0.238
*t*9 C18:1	0.34^Aa^	0.30^Ab^	0.29^Ba^	0.27^Bb^	0.01	< 0.001	0.002	0.337
*t*10 C18:1	0.42^Aa^	0.47^Ab^	0.64^Ba^	0.56^Bb^	0.01	< 0.001	0.284	< 0.001
*c*11 C18:1	0.57^A^	0.60^A^	0.82^Ba^	0.84^Bb^	0.02	< 0.001	0.027	0.784
*t*11 C18:1	2.38^Aa^	3.41^Ab^	5.41^Ba^	5.87^Bb^	0.22	< 0.001	< 0.001	0.463
*c*9,*c*12 C18:2	2.15^a^	1.93^Ab^	2.22	2.13^B^	0.03	0.008	< 0.001	0.005
*c*9,*t*11 C18:2 (CLA)	1.55^Aa^	1.67^Ab^	2.37^B^	2.58^B^	0.07	< 0.001	< 0.001	0.739
*t*9,*c*11 C18:2 (CLA)	0.032^Aa^	0.036^Ab^	0.036^Ba^	0.031^Bb^	0.001	0.271	0.246	< 0.001
*t*10,*c*12 C18:2 (CLA)	0.007^Aa^	0.008^Ab^	0.008^Ba^	0.009^Bb^	< 0.001	< 0.001	< 0.001	0.609
*t*11,*c*13 C18:2 (CLA)	0.011^Aa^	0.009^b^	0.014^Ba^	0.010^b^	< 0.001	< 0.001	< 0.001	0.043
C18:3*n*-3	0.65^Aa^	0.69^Ab^	0.78^Ba^	0.94^Bb^	0.02	< 0.001	< 0.001	0.010
C18:3*n*-6	0.024^A^	0.026^A^	0.036^Ba^	0.034^Bb^	0.001	< 0.001	0.433	0.006
C18:4	0.025^A^	0.022	0.022^Ba^	0.024^b^	< 0.001	0.107	0.548	< 0.001
C20:0	0.126^Aa^	0.142^Ab^	0.133^Ba^	0.120^Bb^	0.002	< 0.001	0.207	< 0.001
C20:1	0.48^Aa^	0.58^Ab^	0.62^Ba^	1.04^Bb^	0.04	< 0.001	< 0.001	0.101
C20:2	0.14^Aa^	0.18^Ab^	0.23^Ba^	0.25^Bb^	0.01	< 0.001	< 0.001	0.141
C20:3	0.062	0.068^A^	0.067^a^	0.085^Bb^	0.002	< 0.001	< 0.001	0.009
C20:4	0.045^Aa^	0.049^Ab^	0.055^B^	0.057^B^	0.001	< 0.001	0.001	0.287
C20:5	0.048^Aa^	0.068^Ab^	0.078^Ba^	0.092^Bb^	0.003	< 0.001	< 0.001	0.192
C22:0	0.048^Aa^	0.053^Ab^	0.052^Ba^	0.046^Bb^	0.001	0.150	0.766	< 0.001
C22:1	0.040^Aa^	0.044^Ab^	0.044^Ba^	0.052^Bb^	0.001	< 0.001	< 0.001	0.167
C22:2	0.042^Aa^	0.036^Ab^	0.034^Ba^	0.038^Bb^	0.001	< 0.001	0.521	< 0.001
C22:5	0.066^Aa^	0.070^Ab^	0.116^Ba^	0.122^Bb^	0.004	< 0.001	< 0.001	0.049
C22:6	0.049^Aa^	0.066^Ab^	0.073^Ba^	0.085^Bb^	0.002	< 0.001	< 0.001	0.055
C24:0	0.014^a^	0.015^b^	0.014	0.015	< 0.001	0.130	< 0.001	0.181

^A,B^Different superscripts indicate significant differences between diets within sampling time at *p* < 0.05.

^a,b^Different superscripts indicate significant differences between sampling times within diet at *p* < 0.05.

^1^Diet: FO—supplemented with raw fish oil; LTC-FO—supplemented with fish oil after the process of low-temperature crystallization.

^2^SEM—standard error of the mean.

^3^*p* value: D—effect of diet (FO vs. LTC-FO); T—effect of sampling time (14 d vs. 30 d); D × T—interaction between diet and time.

Table 5 shows the statistical analysis of the content of fatty acid groups with different degrees of saturation, carbon chain length, and geometrical position and location of unsaturated bonds. The milk fat of cows supplemented with LTC-FO had a significantly higher (*p* < 0.05) mean content of UFA, MUFA, PUFA, LCFA, LC-PUFA and the mean sum of trans C18:1 isomers, CLA isomers, *n*-3 fatty acids and EPA + DHA, and a significantly lower (*p* < 0.05) mean content of SFA, SCFA and MCFA compared to cows receiving FO. Significant effects of diet (D) and sampling time (T) were found for almost all fatty acid groups analysed (*p* < 0.001). Additionally, the D × T interaction effect had a significant effect on 4 out of the 12 fatty acid groups analysed (*p* value ranging from 0.006 to 0.049).

**Table 5:** Fatty acid groups with varying degrees of saturation, carbon chain length*,* location and geometrical position of unsaturated bonds ($\overline{x }$, g/100 g of total FAs) in milk of cows fed two diets at two sampling times (n = 12 animals/group).

Item^4^	FO^1^		FO-LTC^1^		SEM ^2^	*p*-value^3^		
	**14 d**	**30 d**	**14 d**	**30 d**		**D**	**T**	**D x T**
SFA	63.3^Aa^	61.9^Ab^	60.8^Ba^	58.9^Bb^	0.24	< 0.001	< 0.001	0.087
UFA	34.5^Aa^	36.1^Ab^	37.3^Ba^	39.2^Bb^	0.26	< 0.001	< 0.001	0.698
MUFA	29.6^Aa^	31.2^Ab^	31.1^Ba^	32.7^Bb^	0.18	< 0.001	< 0.001	0.942
PUFA	4.90^A^	4.93^A^	6.14^B^	6.50^B^	0.11	< 0.001	0.057	0.157
SCFA	7.90^A^	7.88^A^	6.83^B^	6.68^B^	0.09	< 0.001	0.249	0.346
MCFA	43.9	43.5^A^	43.8^a^	42.4^Bb^	0.12	< 0.001	< 0.001	0.006
LCFA	46.0^Aa^	46.7^Ab^	47.5^Ba^	49.1^Bb^	0.19	< 0.001	< 0.001	0.010
LC-PUFA	0.45^Aa^	0.54^Ab^	0.65^Ba^	0.73^Bb^	0.02	< 0.001	< 0.001	0.556
Σ trans-C18:1	3.14^Aa^	4.19^Ab^	6.34^Ba^	6.69^Bb^	0.23	< 0.001	< 0.001	0.010
Σ CLA	1.60^Aa^	1.72^Ab^	2.43^B^	2.63^B^	0.07	< 0.001	< 0.001	0.616
EPA + DHA	0.097^Aa^	0.134^Ab^	0.151^Ba^	0.176^Bb^	0.004	< 0.001	< 0.001	0.049
Σ* n*-3	0.84^Aa^	0.91^Ab^	1.07^Ba^	1.27^Bb^	0.03	< 0.001	< 0.001	0.289

^A,B^ Different superscripts indicate significant differences between diets within sampling time at *p* < 0.05.

^a,b^ Different superscripts indicate significant differences between sampling times within diet at *p* < 0.05.

^1^Diet: FO—supplemented with raw fish oil; LTC-FO—supplemented with fish oil after the process of low-temperature crystallisation.

^2^SEM—standard error of the mean.

^3^*p*-value: D—effect of diet (FO vs. LTC-FO); T—effect of sampling time (14 d vs. 30 d); D × T— interaction between diet and time.

^4^SFA—saturated fatty acids, UFA—unsaturated fatty acids, MUFA—monounsaturated fatty acids, PUFA—polyunsaturated fatty acids, SCFA—short-chain fatty acids (FAs with C4–10), MCFA—medium-chain fatty acids (FAs with C12–16:0), LCFA—long-chain fatty acids (FAs ≥ C17), LC-PUFA—long-chain polyunsaturated fatty acids (PUFA ≥ C20), Σ trans-18:1—sum of C18:1 *trans*-9, *trans*-10, and *trans*-11, Σ CLA—sum of isomers C18:2 *cis-*9*trans-*11, *trans-*10*cis-*12, *trans-*11*cis-*13, and *trans-*11*trans-*13, EPA + DHA—sum of eicosapentaenoic acid (EPA, C20:5) and docosahexaenoic acid (DHA, C22:6), Σ *n*-3 – sum of C18:3 *cis*-9,12,15, C18:4 *cis*-6,9,12,15, C20:5 *cis*-5,8,11,14,17, C22:5 *cis*-7,10,13,16,19, and C22:6 *cis*-4,7,1

## Discussion

The biological value of fish oil is determined by the content of *n*-3 PUFAs. To increase the concentration of PUFAs in fats, several methods have been developed. In our experiment, the non-complicated method of LTC, which does not require specialised apparatus was used. The LTC method exploits differences in the crystallisation point of individual FAs at temperatures below 0 °C. At low temperatures, SFAs and MUFAs are more prone to crystallisation compared to PUFAs, including *n* − 3 FAs, which remain in the liquid phase^33^. Previous studies have reported that the concentration of PUFAs can be affected by the use of different organic solvents and temperatures^24,34^. Bodkowski et al.^23^ conducted a study to optimize the LTC process for fish oil from sprat-herring waste, using various organic solvents, freezing points, and oil to solvent mass ratios. The authors suggested using acetone as a solvent with an oil-to-solvent ratio of 1:2.5 and a freezing temperature of − 70 °C. These conditions were employed in the experiment (Fig. 1).

Omega-3 LC-PUFAs are chemically unstable and highly susceptible to oxidation due to their high number of double bonds and their geometrical position within the FA chain. The oxidation process results in an unpleasant taste and odour, as well as the formation of toxic substances and unfavourable changes in the configuration of the FAs from cis to trans^35^. To reduce the adverse oxidative changes in mackerel oil enriched in *n*-3 PUFAs, Patkowska-Sokoła et al.^25^ used α-tocopherol as an antioxidant and found a slight decrease in EPA and DHA levels and a slight increase in peroxide and anisidine levels after 4 weeks. In order to limit the adverse oxidative changes in our study, an analogous amount of alpha-tocopherol as antioxidant and stored both preparations (LTC-FO, FO) under identical conditions were used. In order to facilitate the use of LCT-FO and FO in cattle feeding, both formulations were sprayed on the carrier Humokarbowit, which is characterised by high sorptive capacity and antioxidant properties and is suitable for livestock due to its biostimulatory and prophylactic properties^14,36^.

Due to the lack of reports in the available literature on the use of LC-PUFA-enriched fish oil in ruminant diets, the discussion section compares the results obtained in the present study with studies on dietary supplementation with different amounts of fish oil.

In our experiment, the addition of LTC-FO resulted in a lower intake of DM compared to FO. A decrease in DMI with increasing levels of fish oil in the ration of cows was also observed by other authors. In the study by Donovan et al.^16^ at 1, 2 and 3% fish oil addition, DMI was 29.0, 23.5 and 20.4 kg/d, respectively. In the experiment of Keady et al.^13^ in the groups receiving 150, 300 and 450 g/d of fish oil, silage dry matter intake was 10.2, 9.2 and 7.9 kg/d, respectively. Also Kairenius et al.^8^ reported that after rumen administration of 75 and 300 g/d of fish oil, intake of silage and total DM was 10.8 and 18.9 kg/d, and 8.25 and 16 kg/d, respectively.

In our study, supplementation of dairy cows' diets with LTC-FO compared to FO had no effect on milk production, lactose content and yield and protein content, only a decrease in fat content and yield and protein yield in milk was found (Table 3). The results of other authors' studies on the effects of different doses of fish oil in the feeding of cows on milk yield, protein and lactose content were inconclusive^8,13,16^. In most studies, fish oil supplementation, especially in the unprotected form, typically reduces milk fat synthesis in lactating cows. Donovan et al.^13^ showed that fat content and yield decreased linearly with increasing fish oil dose from 1 to 3%. In the study by Kairenius et al.^8^, fish oil supplementation at levels of 75, 150 and 300 g/day reduced milk fat content (by 5.6%, 19.9% and 30.1%, respectively) and fat yield (by 5.2%, 15.1% and 40.6%, respectively). Rego et al.^37^ observed decreases in milk fat content of 4.9 and 11.4% and fat yield of 140 and 340 g/day, respectively, at 160 and 320 g fish oil supplementation. Diet-induced milk fat depression (MFD) is characterised by a significant reduction in fat content due to changes in rumen metabolic pathways, with no change in yield or other milk constituents^38^. For diets containing marine supplements, the biohydrogenation (BH) theory has been proposed, which attributes the reduction in milk fat to the formation of specific FA intermediates, which after absorption in the duodenum and transfer to the mammary gland, inhibit milk fat synthesis^38–40^. Changes in the FA profile associated with FO-induced MFD clearly indicate that the decrease in milk fat content to inhibition of de novo FA synthesis rather than preformed FA uptake in the mammary gland^41,42^. A typical phenomenon associated with the use of fish oil supplements in cattle feed is an increase in trans-C18:1 isomers in milk fat^38,39,43^. Numerous studies have shown that FO-induced MFD is also associated with a reduced proportion of 18:1 *cis*-9 in milk fat^44,45^. In our study, as a result of supplementation with LTC-FO compared with FO, the proportion of C18:1 *trans*-10 and *trans*-11 in milk fat increased and the proportion of C18:1 *cis*-9 decreased, which may explain the lower fat content in the LTC-FO group.

Milk fat synthesis depends on two general sources of FAs. The short- (SCFA) and medium-chain (MCFA) FAs (C4–C14) and half of the C16 FAs are synthesised de novo in the mammary gland, while FAs > 18-carbon are transferred from preformed blood triglycerides^46^. As a result of feeding fish oil to ruminants, changes in rumen BH pathways occur towards reduced content of milk fat SFA and increased LC-PUFA^8,47^. Fish oil supplementation decreased the proportion of 4–16-carbon FAs in milk^38,39^, which may be explained by the inhibitory effects of increased long-chain FAs availability on genes involved in lipid metabolism in the mammary gland^48^. Previous studies have shown that fish oil results in a dose-dependent decrease in the proportion of FAs synthesised de novo in lactating cows^8,13,37^.

In our research, the levels of butyric acid (C4:0), caproic acid (C6:0), caprylic acid (C8:0) and capric acid (C10:0) in the SCFA group decreased as a result of supplementing cows' diets with LCT-FO vs. FO (Table 4). In the experiment by Kairenius et al.^8^ the levels of C4:0, C6:0, C8:0 and C10:0 were lower when fish oil was added at 150 and 300 g/d than at 75 g/d. In the studies by Keady et al.^16^, Donovan et al.^13^ and Rego et al.^37^, a greater decrease in C6:0, C8:0 and C10:0 was observed with increasing fish oil dose. Conversely, in our study in the MCFA group, the lauric acid (C12:0) and palmitic acid (C16:0) content decreased and the palmitoleic acid (C16:1 *cis*-9) content increased (Table 4), which is confirmed by the results of the other studies as the level of FO in the dose increases^8,37^.

Under normal conditions, stearic acid (C18:0) is the major FA leaving the rumen during the BH process^49^. Supplementation of ruminant diets with marine lipids inhibits the final step of BH to C18:0 in the rumen^50^, which reduces its uptake by the mammary gland and consequently reduces the synthesis of oleic acid (18:1 *cis*-9) by delta(9)-desaturase^51^. In addition, the use of marine lipids results in an increase in trans-18:1 isomers in milk fat, which have a higher melting point than cis-C18:1 isomers^8,21^. As a result of LTC-FO vs. FO supplementation, the content of C18:1 *cis*-9 in milk fat decreased and the content of C18:1 *trans*-10 and *trans*-11 increased (Table 4). In the experiment by Shingfield et al.^52^, fish oil increased the content of trans FA in milk, probably due to incomplete BH of unsaturated 16- to 22-carbon FA in the rumen. In vitro studies have shown that both EPA and DHA inhibit the reduction of 18-carbon unsaturated FA to 18:0, resulting in the accumulation of trans-18:1 intermediates^53,54^. Loor et al.^21^ and Shingfield et al.^55^ reported that most of the increase in total milk trans FA content due to fish oil supplementation was associated with a specific enrichment of trans-18:1 isomers (Δ^8–12^). The linear increase in the content of C18:1 isomers of the configurations *trans*-10 and *trans*-11 (TVA) with increasing fish oil dose was observed in the study by Kairenius et al.^8^, TVA in the research by Donovan et al.^13^ and total trans-C18:1 isomers in the experiment by Keady et al.^16^.

Marine lipids with high content of 20- and 22-carbon PUFA have often been used to inhibit the saturation of 18:1 *trans*-11 with the ultimate aim of increasing C18:2 *cis*-9,*trans*-11 in milk^8,56^. In our experiment a higher level of the CLA isomer *cis*-9,*trans*-11 (RA, rumenic acid) was observed in the LTC-FO group compared to the FO group (Table 4). An increase in RA content was also observed in the studies by Kairenius et al.^8^ (1.03 vs. 2.15 g/100 g total FA, 75 vs. 150 g/d fish oil), and Donovan et al.^13^ (1.58 vs. 2.23 g/100 g total FA, 1 vs. 2% fish oil).

Supplementation with LTC-FO resulted in an increase in the content of α-linoleic acid (ALA, C18:3 all-*cis*-9,12,15; *n* − 3) and γ-linoleic acid (GLA, C18:3 all-*cis*-6,9,12; *n* − 6) compared to FO supplementation (Table 4). This is consistent with previous studies, such as Keady et al.^16^, where GLA content increased with higher levels of fish oil intake (150–300 g/d). Kairenius et al.^8^ also found an increase in ALA content with the highest dose of fish oil. In contrast, Donovan et al.^13^ found that the higher level of fish oil in cows’ feed had no effect on the content of ALA, and caused a decreased in GLA.

The supplementation of LTC-FO vs. FO resulted in an increase in the content of most long-chain PUFAs (≥ 20-carbon) found in milk fat. These include eicosanoic acid (C20:1 *cis*-11), eicosadienoic acid (EDA, C20:2 *cis*-11,*cis*-14; *n* − 6), dihomo-γ-linolenic acid (DGLA, C20:3 all-*cis*-8,11,14; *n* − 6), and arachidonic acid (AA, C20:4 all-*cis*-5,8,11,14; *n* − 6) (Table 4). Previous research has shown that increasing the amount of fish oil in the diet leads to an increase in the milk fat content of C20:1, C20:2, C20:3 and C20:4^8^, C20:1 and C20:4^13^, and C20:1, C20:2 and C20:4^16^.

Eicosapentaenoic acid (EPA, C20:5 all-*cis*-5,8,11,14,17; *n* − 3) and docosahexaenoic acid (DHA, C22:6 all-*cis*-4,7,10,13,16,19; *n* − 3) are typically present in very low amounts in milk due to their absence or minimal levels in traditional dairy cow diets^13,20^. The transfer of dietary EPA and DHA to milk fat in lactating cows is very low (< 4%)^19^, reflecting extensive BH by rumen bacteria^21,52^. The low transfer may also be a result of the partitioning of EPA and DHA into plasma lipid fractions that are less available to the mammary gland, such as triglycerides and non-esterified fatty acids^57^. Additionally, after absorption, EPA and DHA are incorporated into cholesterol esters and phospholipids that have a low affinity for lipoprotein lipase in the mammary endothelium^58^. The BH process activity decreased when fish oil was added to the animals' feed rations. This is likely due to the toxic effects of PUFAs on certain rumen bacteria^20,40,52^. Previous studies have shown that supplementing with fish oil increases the content of milk C20:5, C22:5, and C22:6 in a dose-dependent manner^8,13,16^. The effective transfer of these fatty acids is greater when they are delivered in a way that bypasses rumen metabolism^56^. In our experiment, adding LTC-FO instead of FO resulted in an increase in milk fat EPA, DHA, and docosapentaenoic acid (DPA, C22:5 all-*cis*-7,10,13,16,19; *n* − 3) (Table 4). Rego et al.^37^ conducted an experiment where they found that the content of EPA in milk fat increased by 2.6- and 4.7-fold (from 0.007 to 0.18 and 0.33 g/100 g FA, respectively), and DHA by 2.8- and 7.2-fold (from 0.06 to 0.17 and 0.43 g/100 g FA, respectively), for treatments of 160 and 320 g/d of fish oil. Similarly, Keady et al.^16^ demonstrated that EPA content was significantly higher with fish oil supplementation of 450 g/d compared to 150 or 300 g/d. Kairenius et al.^8^ found that milk fat from cows supplemented with 300 g/d fish oil had significantly higher contents of EPA, DHA, and DPA than that supplemented with 75 or 150 g/d.

When analyzing changes in the content of FA groups with varying degrees of saturation and carbon chain length, in the LTC-FO fed cows a decrease in the proportion of SFAs in milk fat was observed, including short-chain SCFAs, and an increase in the proportion of UFAs, PUFAs, and LC-PUFAs (Table 5). This finding is consistent with other reports. Donovan et al.^13^ reported a decrease in the proportion of SFAs and SCFAs, and an increase in the proportion of UFAs in cow's milk as a result of increased addition of fish oil. Similarly, Kairenius et al.^8^ observed a linear decrease in the proportion of SFAs and an increase in PUFAs in milk fat with an increase in the level of fish oil in cows' diet. Rego et al.^37^ showed that increasing dietary fish oil intake led to a decrease in total SFAs and the proportion of short-chain fatty acids (C4:0 to C12:0), while the proportion of medium-chain fatty acids (C14:0 to C16:1) remained unchanged.

As a result of LTC-FO compared to FO supplementation, total CLA, trans-18:1 isomers, *n*-3 FAs and EPA + DHA in milk fat increased (Table 5). As in our study, Kairenius et al.^8^ found an increase in total CLA and trans isomers in milk fat and Donovan et al.^13^ found an increase in total *n* − 3 FAs with an increase in fish oil supplementation in cows' diets. Conversely, in the study by Rego et al.^37^, an increase in fish oil supplementation in cows' diets was associated with an increase in EPA + DHA in milk fat.

Functional food is the fastest growing sector of the global food market^59^. Cow's milk is a strategic commodity and a staple food. Modification of the FA profile by supplementation with fish oil or its combination with plant oils is a good dietary strategy to reduce the intake of SFAs in favour of healthier FAs (e.g. *n* − 3 LC-PUFA, CLA)^8,14,60,61^. Studies conducted in patients with myocardial infarction, metabolic syndrome and vascular disease describe benefits and suggest that milk enriched with *n* − 3 LC-PUFA (EPA and DHA) may be useful as a dietary supplement to control risk factors^62^.

In 2022 the EU average of milk consumption was about 53.4 kg per person, which is about 140 g/d^63^. Consuming an equivalent amount of milk from LTC-FO-supplemented cows can provide a person with 388 mg EPA and 360 mg DHA, while from FO-supplemented cows 297 mg and 294 mg, respectively. Recommendations for daily EPA + DHA intake depend, among other factors, on age (children, adult, elderly), gender (men, women), health status (for cardiovascular health), physiological status in women (pregnant, lactating), and range from 250 to 670 mg/d. For healthy adults (19–57 years), the minimum intake should be 500 mg/d^64,65^. This amount of EPA + DHA in a person's diet can provide an intake of approximately 95 g/d of milk from cows supplemented with LTC-FO.

## Conclusion

Supplementing the rations of lactating dairy cows with the developed additive LTC-FO, compared to the same amount of FO, resulted in a decrease in milk fat content and yield. The decrease in milk fat secretion was accompanied by a lower proportion of saturated fatty acids, mainly short-chain (C4–10) and medium-chain (C12 and C16). On the other hand, the content of biologically active FAs (i.e. EPA, DHA CLA, ALA and TVA) increased significantly in milk fat. Therefore, fish oil enriched with *n* − 3 PUFAs, as a result of a non-complicated and not requiring special equipment low temperature crystallisation process, can be a more efficient feed additive than raw fish oil and may serve as a practical way to modify the FA composition of milk fat.

## Data and model availability

The data supporting the findings of this study are available from the corresponding author upon reasonable request.
